# Evolutionary Trajectory for the Emergence of Novel Coronavirus SARS-CoV-2

**DOI:** 10.3390/pathogens9030240

**Published:** 2020-03-23

**Authors:** Saif ur Rehman, Laiba Shafique, Awais Ihsan, Qingyou Liu

**Affiliations:** 1 State Key Laboratory for Conservation and Utilization of Subtropical Agro-bioresources of Guangxi University, Nanning 530005, China; Saifurrehman4337904@gmail.com (S.u.R.); laibazoologist@gmail.com (L.S.); 2 Department of Biosciences, COMSATS University Islamabad, Sahiwal Campus 57000, Pakistan; 3 College of Pharmacy, South Central University for Nationalities, Wuhan 430074, China

**Keywords:** SARS-CoV-2, SARS-CoV, phylogeny, genomic structure, evolutionary strategies, mutations, recombination or reassortment

## Abstract

Over the last two decades, the world experienced three outbreaks of coronaviruses with elevated morbidity rates. Currently, the global community is facing emerging virus SARS-CoV-2 belonging to *Betacoronavirus*, which appears to be more transmissible but less deadly than SARS-CoV. The current study aimed to track the evolutionary ancestors and different evolutionary strategies that were genetically adapted by SARS-CoV-2. Our whole-genome analysis revealed that SARS-CoV-2 was the descendant of Bat SARS/SARS-like CoVs and bats served as a natural reservoir. SARS-CoV-2 used mutations and recombination as crucial strategies in different genomic regions including the envelop, membrane, nucleocapsid, and spike glycoproteins to become a novel infectious agent. We confirmed that mutations in different genomic regions of SARS-CoV-2 have specific influence on virus reproductive adaptability, allowing for genotype adjustment and adaptations in rapidly changing environments. Moreover, for the first time we identified nine putative recombination patterns in SARS-CoV-2, which encompass spike glycoprotein, RdRp, helicase and ORF3a. Six recombination regions were spotted in the S gene and are undoubtedly important for evolutionary survival, meanwhile this permitted the virus to modify superficial antigenicity to find a way from immune reconnaissance in animals and adapt to a human host. With these combined natural selected strategies, SARS-CoV-2 emerged as a novel virus in human society.

## 1. Introduction

The global community is at the peak of emerging bugs, even though the earlier scares of zoonotic viruses were not retained. The re-emergence of viral agents is a great threat and challenge for the global health community [1]. The global community has witnessed that over the last two decades, the world has experienced three outbreaks of coronaviruses with elevated morbidity rates. In December 2019, cases of mysterious pneumonia with unknown etiology were reported in Wuhan, Hubei, a province of China, which got the attention of the world [2]. Researchers and the Chinese government responded swiftly, and after deep etiological and sequencing investigation, the International Committee on Taxonomy of Viruses entitled it as severe acute respiratory syndrome coronavirus 2 (SARS-CoV-2) [3].

**Figure 1:**
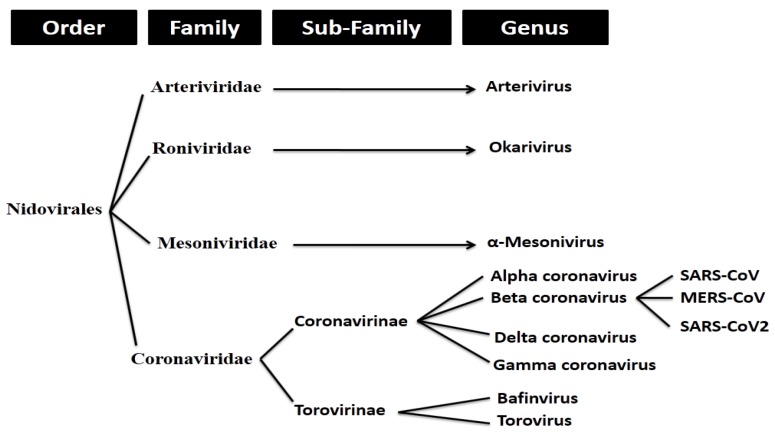
Classification of coronaviruses.

The SARS-CoV-2 belongs to *Betacoronavirus*, a member of the subfamily *Coronavirinae* having four genera: *Alphacoronavirus, Betacoronavirus, Gammacoronavirus,* and *Deltacoronavirus* in family *Coronaviridae*, categorized in the order *Nidovirales* (Figure 1).

Generally, CoVs are broadly distributed among humans, birds and other mammals, usually causing hepatic, enteric, neurologic and respiratory syndromes [4,5]. Four (229E, OC43, NL63 and HKU1) out of six human disease-causing CoVs are widespread, and in immune-competent individuals they normally cause common cold symptoms [6]. Two other strains that were linked with fatal illness were zoonotic in origin, including severe acute respiratory syndrome coronavirus (SARS-CoV) and Middle East respiratory syndrome coronavirus (MERS-CoV) [7].

In 2002–2003, the outbreak of severe acute respiratory syndrome occurred due to SARS-CoV in the Guangdong Province of China and quickly became pandemic to twenty-seven countries, infecting 8098 people with 774 deaths and was declared the first endemic of the 21st century [8]. A decade later in 2012, MERS-CoV caused a severe respiratory disease that emerged in the Middle East with 2494 confirmed human infection cases and 858 deaths [9]. In both epidemics, bats were identified as the original source of SARS and MERS-CoVs. The rate of human-to-human transmission of SARS-CoV-2 appears higher than earlier outbreaks of CoVs via cough and/or sneezing droplets emitted from an infected person. SARS-CoV-2 has appeared more transmissible but less deadly than SARS-CoV. To date worldwide, 194,029 confirmed cases of human infection and 7873 deaths across 164 countries have been recorded [10].

In natural populations, mutations, recombination, and reassortment are the strategic evolutionary process considered for genetic diversity. The high incidence of homologous RNA recombination is one of the most fascinating features of CoVs replication [11,12,13,14]. Kottier et al. reported the first experimental-based recombination evidence for avian infectious bronchitis virus (IBV) [15], although additional studies have also concluded that IBV evolves through recombination [16,17,18,19,20,21]. Moreover, murine hepatitis virus (MHV) evolution through recombination was also practically confirmed [22]. This encouraged exploration of the probable role of recombination in the SARS-CoV emergence. The current condition might appear as a vulnerable factor for severe disease and may impose serious health threats to the human. Due to wide distribution with the increasing prevalence of CoVs, frequent genomes recombination, large genetic diversity and high human-animal interface behavior, CoVs might be emerged from time-to-time in humans due to occasional spillover and recurrent cross-species infectious events [7,23].

As an emerging virus, very limited information is available to describe the genetic diversity, evolutionary ancestors and possible routes of transmission of SARS-CoV-2 from the natural reservoir to humans. This study aimed to track the evolutionary ancestors of SARS-CoV-2 and different evolutionary strategies (mutations, recombination or reassortment) that were genetically adapted by the novel coronavirus.

## 2. Results

### 2.1. Whole Genome-Based Molecular Phylogenetic Analysis of Coronavirus

**Figure 2:**
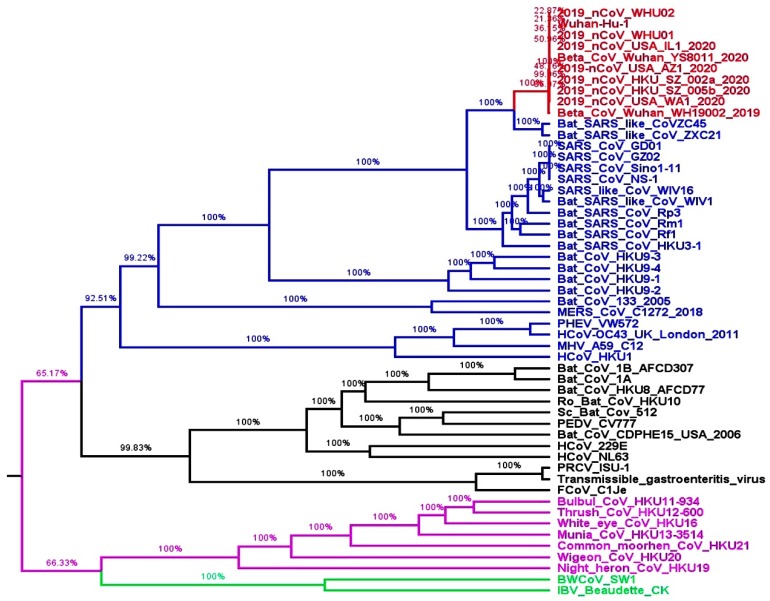
Evolutionary phylogenetic tree analysis of Coronaviruses: whole-genome sequences based on the phylogenetic tree of CoVs was constructed with the maximum-likelihood method using BEAST with GTR+I+G as the nucleotide substitution model with an applied posterior probability value of 0.5. Branches with different colors represent different genera of Coronaviruses; black, alpha coronavirus, blue, beta coronavirus; red, SARS-CoV-2; green, delta coronavirus; purple, gamma coronavirus.

It is the utmost priority of the scientific community to minimize the public health risk through tracing the origin and natural inhabitants of SARS-CoV-2 to restrict human-to-human and cross-species transmission. To understand the genetic diversity relationship and potential origin of SARS-CoV-2 with the other members of coronavirinae, we have performed molecular phylogenetic analysis with a number of CoVs whole-genome sequences obtained from National Center for Biotechnology Information (NCBI) (Table S1). The phylogenetic tree results demonstrate that all the CoVs responsible for the outbreak of concentrated pneumonia belong to the genera *Betacoronavirus* (Figure 2). All the SARS-CoV-2 clade grouped with the cluster of SARS/SARS-like CoVs, with bat CoVs HKU9-1, HKU9-2 HKU9-3 and HKU9-4 as an immediate ancestor (Figure 2). The interior shared neighbors include SARS-CoV NS-1, SARS-CoV Sino1-11, SARS-CoV GZ02 and SARS-CoV GD01, and they were the human-infecting CoVs (Figure 2). The whole genome-based phylogenetic analysis presented that two Bat SARS-like CoVs (ZXC21 and ZC45) were the closest relatives of SARS-CoV-2. Most of the inner and outer joint neighbors of SARS-CoV-2 were found to have bats as their natural reservoir including Bat SARS-CoV WIV1 in *Rhinolophus sinicus*, Bat SARS-CoV HKU3-1, and Bat CoV HKU9-3 in Rousettus bats. Consequently, the bat would be the convenient native host of SARS-CoV-2, thus the probable intermediate host for the transmission cascade used by SARS-CoV-2 from bats to humans would be the same as that used by other SARS-CoV.

### 2.2. Comparative Genomics of Wuhan-Hu-1-CoV and SARS CoV

**Figure 3:**
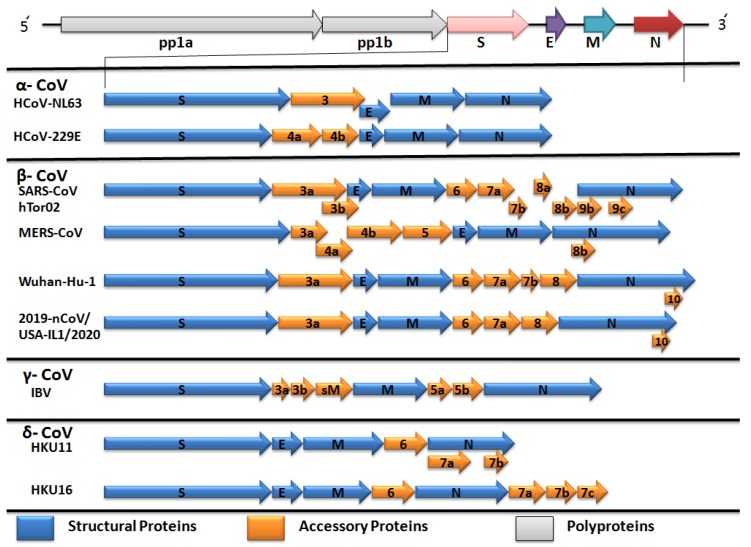
Genomic and gene view of four coronaviruses genera.

CoVs genome is comprised of single-stranded positive-sense RNA with 5’-cap and 3´-poly-A tail (Figure 3). At the 5´ end, non-structure protein including poly-proteins pp1a and pp1b are directly translated from the genomic RNA strand. Other structure proteins are envelope (E), nucleocapsid (N), membrane (M) and spike (S) proteins. In addition, CoVs encoded some special accessory proteins like 3a/b, 4a/b, 5, 6, 7a/b etc. proteins (Figure 3).

**Figure 4:**
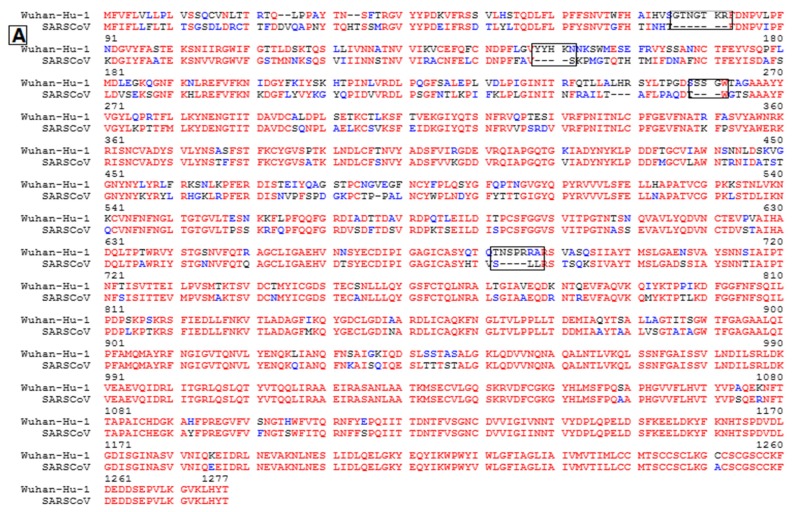

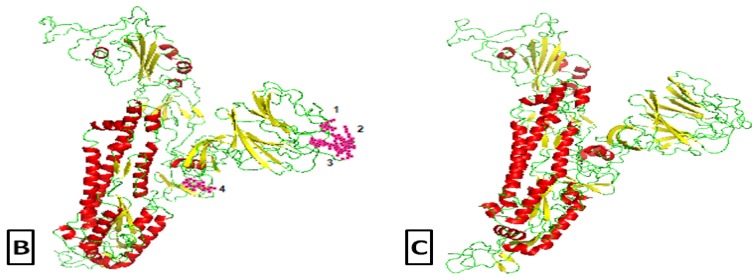
Comparison of Spike (S) protein amino acid residue sequence of Wuhan-Hu-1-CoV and SARS-CoV; (**a**): Wuhan-Hu-1-CoV (Wuhan seafood market pneumonia virus) and SARS-CoV (GZ02) amino acid residue multiple sequence alignment with hierarchical clustering (**b**,**c**): Prediction of S protein structure by using homology protein modeling (**b**) (Wuhan-Hu-1-CoV) and (**c**) (SARS CoV GZ02). Secondary structure selection by representing color includes: red, helix; yellow, sheets; green, loops; pink, insertions.

Wuhan-Hu-1-CoV in the phylogenetic tree was uniquely positioned with SARS/SARS-like CoVs that share a common ancestor which resembled bat coronavirus HKU9-1, HKU9-2 HKU9-3 and HKU9-4. During the course of evolution, various recombinant events possibly obscure the path substantially by the patterns of genomic homologous diversity. Thus, we compared the E, M, N and S genomic regions of Wuhan-Hu-1-CoV as representative of SARS-CoV-2, SARS, and MERS-CoV (Figures S1–S4). Wuhan-Hu-1-CoV genome had more sequence homology with SARS-CoV (Table 1, Figure 4) as compared to MERS-CoV. Although high genetic diversity was found between Wuhan-Hu-1-CoV and SARS-CoV (Table 1) in the E, M, N and S genes, low sequence homology between Wuhan-Hu-1-CoV and MERS-CoV was observed (Figures S1–S4).

Examining the envelop (E) protein disclosed that the sequence conservation of Wuhan-Hu-1-CoV in view of SARS-CoV was more than MERS CoV (Figure S1). Wuhan-Hu-1-CoV shared 93% amino acid sequence homology with 7% genetic variation in the E protein with the SARS-CoV (Table 1).

Furthermore, the amino acid sequence of the membrane (M) protein of Wuhan-Hu-1-CoV, SARS-CoV, and MERS-CoV were compared (Figure S2). In Wuhan-Hu-1-CoV, about 92% conservation of amino acid sequences and 17 mutations (8%) with respect to SARS-CoV were observed (Table 1).

Further, we analyzed the nucleocapsid (N) protein, which is a more abundant protein in CoVs. With no exception, about 93% of the amino acid sequence identity of the N protein for Wuhan-Hu-1-CoV with SARS-CoV (Table 1) with 7% genetic variations was found, while a less conserved sequence percentage was found with MERS-CoV (Figure S3). The conserved nature of SARS-CoV-2 along with other CoVs was an important factor to trace the evolutionary pathway of the CoV and would be important to limit the outbreak of pneumonia-related viruses. In CoVs, the N protein was crucial for RNA transcription and viral assembly disrupting the host cell and is also important to evaluate the virus-host adaptation and drug design.

Further, we compared spike glycoprotein (S) protein of Wuhan-Hu-1-CoV, SARS-CoV and MERS-CoV (Figure S4). It was observed that during the viral infection, S protein underwent several drastic changes. The S protein of Wuhan-Hu-1-CoV was more prone to mutations; particularly, the amino acid sequence represented ~19% alteration with four major insertions and ~81% homology in contrast to SARS (Table 1, Figure 4a,b). Additionally, we found that the Wuhan-Hu-1-CoV S protein was vulnerable to mutations, especially in spike protein-cell receptor interface-associated amino acids.

SARS-CoV used a receptor-binding domain that stretched between 306–527 amino acid sequences. We compared the receptor-binding domain of SARS-CoV and Wuhan-Hu-1-CoV and we found that 73% of conserved amino acid regions were observed in Wuhan-Hu-1-CoV (Figure S5). In the meantime, similar conservation patterns of the amino acid were also found in the binding receptor motif extended 424–494 amino acid residues used by SARS-CoV to bind human ACE2 (Figure S6). It was suggested that a significant affinity of Wuhan-Hu-1-CoV with ACE2 imposed high public health risk for humans by transmission through the S protein-ACE2 binding channel. Subsequently, homology protein modeling was used to predict the S protein structure of Wuhan-Hu-1-CoV via the structure of SARS-CoV spike glycoprotein (PDB: c5xlrC) (Figure 4b,c).

### 2.3. Recombination Events in Newly Emerged Coronavirus

**Figure 5:**
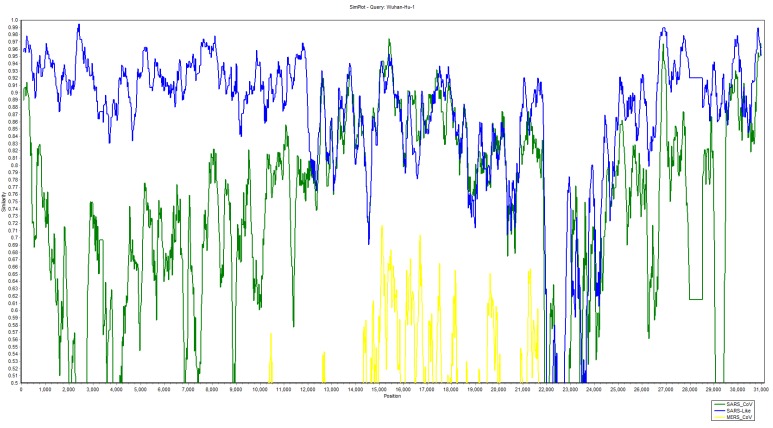
Similarity plot of Wuhan-Hu-1-CoV with other Coronaviruses (Blue, Bat SARS Like-CoVs W1V, ZXC21, ZC45; green, Bat SARS-CoVs GZ02, RF1; and yellow, MERS-CoV).

Nine regions in complete genome nucleotide sequences of Wuhan-Hu-1-CoV were detected as putative recombinant regions and our recombination detection program (RDP) analysis suggested that Wuhan-Hu-1-CoV could be a recombinant of SARS (GZ02, Rf1), SARS-like (ZXC21, ZC45, W1V1) and MERS-CoVs (Table 2). The PHI-test provided significant evidence of recombination (*p*-value < 0.00001). Moreover, the similarity plot showed that the 5-genomic region of Wuhan-Hu-1-CoV shared substantially higher similarity with SARS-like CoVs, while the 3-genomic region shared a mixture of SARS and SARS-like CoVs nucleotide sequence (Figure 5). Taken together, our study found that most of the recombination events occurred in the spike glycoprotein motif of Wuhan-Hu-1-CoV, mostly towards the 5´end of the S gene. Only a single recombination event was identified in RNA-dependent RNA polymerase, helicase, and ORF3a (Table 2).

## 3. Discussion

SARS-CoV-2 is a novel emerging contagious agent that found a way into human civilization. The outbreak of SARS-CoV-2 is the third pandemic of the 21st century and the situation is still ongoing. The prediction of Fan et al. [24] that a future SARS or MERS-like CoVs epidemic would emerge in China with a probable bat source became reality when the first case of concentrated viral pneumonia was reported on December 30, 2019 in Wuhan city of China [25]. Later on, the novel coronavirus designated as SARS-CoV-2 was found responsible for the viral outbreak of pneumonia in Wuhan [26].

Generally, emerging and re-emerging viral infections belong to the RNA family of viruses since these viruses have high mutation rates that lead to eminent environmental adaptation with rapid evolution [27]. To date, very little knowledge is available about SARS-CoV-2. To understand the genetic diversity relationship and potential origin of SARS-CoV-2, our molecular phylogenetic analysis predicted that SARS and SARS-like CoVs were the ancestors of SARS-CoV-2. Two bat SARS-like CoVs (ZXC21 and ZC45) were the closest relatives of SARS-CoV-2 (Figure 2). Consequently, we found that the bat would be the convenient native host of SARS-CoV-2. Previously, it was found that several bat CoVs were able to cause infection in humans without any intermediate host [28,29].

Rapid sequencing of SARS-CoV-2 provided an opportunity for the research community to look into its genetic diversity, developing diagnostic tests and ultimately helping with vaccine production. The whole-genome sequence of SARS-CoV-2 retained ~80% nucleotide homology with SARS epidemic viruses. All the structural proteins were well conserved except for spike glycoprotein that showed a high rate of mutation in SARS-CoV-2 [30,31]. Our results demonstrated that compared with SARS-CoV, the SARS-CoV-2 shares ~81% amino acid similarity in spike (S) protein (Table 1, Figure 4), which represented less conserved patterns of S protein than other CoVs like HKU3-CoV [32]. Through deep receptor-binding domain (RBD) analysis of SARS-CoV (amino acids), the SARS-CoV-2 RBD was 73% preserved comparatively to the pandemic RBD (Figure S5). This conservation pattern of RBD placed the SARS-CoV-2 between HKU3-4 (62.7% conserved), a bat virus that was not capable of using the human ACE2 receptor, and the divergent bat CoV rSHC014 (80.8%), a spike known to use the human ACE2 receptor for entrance [29,33]. Moreover, the binding free energies for the S-protein to human ACE2 binding complexes were calculated and the binding free energy for the Wuhan-Hu-1-CoV S-protein increased by 28 kcal mol^–1^ when compared to the SARS-CoV S-protein binding, representing more binding affinity to the human ACE2 receptor [34].

Moreover, a recent study revealed that a polybasic cleavage site was present at the S1 and S2 junction of SARS-CoV-2 that effectively allowed cleavage by furin and the other protease and took part in viral host range and infectivity [35], whereas these polybasic cleavage sites in other human beta-corona viruses have not been detected [36]. Experimental investigation of Follis et al. with SARS-CoV demonstrated that furin cleavage site insertion at the S1-S2 junction increases cell-cell fusion [37]. Additionally, an effective cleavage site in the MERS-CoV spike motif empowers bat MERS-like CoVs to infect human cells [38]. On the other hand, in avian influenza viruses, quick replication and diffusion effectively acquired polybasic cleavage sites in the hemagglutinin (HA) protein, which served a similar function to that of the coronavirus spike protein. In CoVs, insertion or recombination facilitates acquisition of transforming low-pathogenicity into highly pathogenic forms for polybasic cleavage sites [39]. So far sampled pangolin beta-corona viruses and the bat beta coronaviruses do not have polybasic cleavage sites. CoVs could have adopted a natural evolutionary mechanism to mutate and to attain the polybasic cleavage site because the virus must have both the mutations and polybasic cleavage site for appropriate human ACE2 receptor binding. For this purpose, it required a large population density for natural selection to attain an ACE2-encoding gene that is akin to the human ortholog [40,41]. The recent study of Peng et al. revealed that might it be possible that SARS-CoV-2 ancestors jumped into humans, getting the genetic features through adaptation and remaining undetected during human-to-human transmission. Once it adapted, these variations became pandemic and sufficiently produced a large number of cases to activate the immune system that identified it [40,41].

Usually viruses adopt different strategies including recombination, mutation and reassortment which facilitate the viruses in getting to equilibrium in the final host. Due to low fidelity of reverse transcriptase and RNA-dependent RNA polymerase, RNA viruses are more vulnerable to point mutations even though the point mutation rates in RNA viruses are approximately 10^−4^ to 10^−5^ [42]. During the 2002 SARS-CoV epidemics, three mutations per RNA in each replication round were estimated (8.26 × 10^−6^ per nucleotide per day) [43]. Often, large population size and high rate of mutations in RNA viruses rapidly adjust genotypes allowing for quick adaptations in a rapidly changing environment. Respectively, mutations have a specific influence on virus reproductive fitness as positive selection drives to fix the positive fitness effects of beneficial alleles, while negative selection removes lethal and deleterious alleles from a population. Together with these selective approaches, the evolutionary routes of virus populations can be figured out across a sequence space [34]. Examining the genetic insight of SARS and Wuhan-Hu-1-CoV presented more than 90% sequence conservation between the E, M and N protein with few numbers of point mutations (Table 1), whereas the higher rate of mutations in the S protein of Wuhan-Hu-1-CoV were also observed and shared ~81% identity (Table 1, Figure 4A). These results were in accordance with the results of Xu et al. and Pradhan et al. [44,45].

Recombination and reassortment became a powerful tool of emerging viruses to get innovative antigenic combinations that might aid the course of cross-species diffusion. The recombination strategy facilitates this mechanism to find a better fraction of sequence space than the mutation, raising the probability of finding a genetic configuration which supports host adaptations [46]. It is important to note that numerous recently emerged RNA viruses which were involved in human diseases exhibited active recombination or reassortment events. Mostly RNA viruses get entry into the new host through the cross-species transmission [47]. The recombination events in viruses are in fact related to discontinuous utilization of RNA polymerase involved in the transcriptional mechanism to make mRNAs. RNA polymerase of viruses must use different RNA prototypes while making negative or positive RNA strands that eventually result in RNA recombination that is either homologous or non-homologous [12]. In RNA viruses, this model of recombination is called the copy-choice model of recombination [13,14]. In CoVs, a high recombination rate has been reported [48]. It might be due to having large genome size, discontinuous transcription, and sub- or fully transcriptionally active genomic length of RNA. The co-infection of two CoVs in same animal or cells can potentially facilitate crossing over. In the recent past, the emergence of new infectious bronchitis virus recombinant (IBV), a new type of CoV in turkeys, was reported. The genome sequence revealed that the S protein gene of this virus was the recombinant of another CoVs [49]. In the S protein, the recombination event is certainly significant as it permits the virus to modify superficial antigenicity to get from the immune reconnaissance into the animals, and then adapt to a human host. We identified nine putative recombination patterns, which encompass, in terms of genes involved, the spike glycoprotein, RdRp, helicase and ORF3a. Six of the nine recombination regions were spotted in the S gene (Table 2). Significantly, in this study each of the recombinant regions were predicted with at least two methods (Table 2) according to the method of Posada. He recommended that one should not be dependent on a single method [50]. These results were in agreement with previous reports where the recombinant event was reported between parent viruses in the avian-like and mammalian-like SARS-CoV evolution [51,52].

When segments of multiple viral genomes infect the same animal or tissue simultaneously, it ultimately results in new viral progeny with a multiple parent genome set. This process is termed as gene reassortment used by viruses for evolution [28]. The literature suggests that a typical RNA influenza A virus has eight ssRNA segments and the assortment occurred among multiple influenza viruses termed as genetic “shift’’ or ‘‘antigenic shift’’ resulted in the change of influenza viral surface glycoprotein’s/neuraminidase. Thus, the sequence of these virus strains diverges widely when host animal cell gets infected by confection and the progeny is developed by reassortment or recombination [27].

Taking this together, we found that SARS-CoV-2 was the descendent of SARS/SARS-like coronaviruses, being a close relative of Bat SARS-like CoVs (ZXC21 and ZC45). We confirmed that mutations in different genomic regions of SARS-CoV-2 have a specific influence on virus reproductive adaptability, allowing genotypes to adjust and quickly adapt in a rapidly changing environment. Moreover, for the first time we identified nine putative recombination patterns in SARS-CoV-2 which were undoubtedly important for evolutionary survival, meanwhile permitting the virus to modify superficial antigenicity to get from immune reconnaissance into animals and then adapting to a human host. With these combined natural selected strategies, SARS-CoV-2 emerged as a novel virus in human society.

## 4. Materials and Methods

For molecular phylogenetic analysis, the whole-genome sequences of 53 viruses including 10 SARS-CoV-2 were retrieved from NCBI through BLASTn search, with Wuhan-Hu-1-CoV being used as reference (Table S1). All the sequences were aligned by using MAFFT (V 7.452) online server [53]. To determine the nucleotides substitution model, the Bayesian information criterion (BIC) value for aligned sequences was determined using jModel Test 2 and the substitution model with minimum BIC values was considered for phylogenetic inference (Table S2) [54]. The whole-genome sequence was considered as a single partition, and three chains of Bayesian analysis were performed by applying the GTR+I+G model of substitution. Reaching the maximum allowed number of generations after discarding burin (270030000), the optimal analyses trees were pooled into a single tree file. Posterior probability values with majority consensus rule were visualized. Figure 3 was used to visualize the best tree and the likelihood phylogram was exported as a picture [55]. Multalin software was used to align and visualized the envelope, membrane, nucleocapsid, and spike glycoprotein regions of SARS-CoV, MERS-CoV and SARS-CoV-2 [56]. The amino acid conservation motifs of the receptor-binding domain (RBD) in SARS-CoV and SARS-CoV-2 genome were traced by performing MUSCLE alignment using MEGAX software. The three-dimensional structures of spike glycoproteins of SARS-CoV2 and SARS-CoV were generated by using an online server Protein Homology/analogY Recognition Engine V 2.0 (Phyre2) [57] and the structure was visualized and marked by using PyMol [58]. To detect the recombination events, whole-genome nucleotide sequences of seven viral strains (Wuhan-Hu-1-CoV; Bat SARS-like including W1V1, ZXC21, ZC45; Bat SARS GZ02, RF1 and MERS) were aligned using ClustalW. Preliminarily, MaxChi and Chimaera algorithms were used to detect the recombination events in the dataset by a recombination detection program (RDP5) [59]. Additionally, bootscan analyses and similarity plots were performed using Simplot 3.5.1 [60] to confirm the RDP-suggested potential recombination events and were analyzed on the whole-genome sequence of Wuhan as a query and Bat SARS-like, SARS and MERS as potential parental sequences (Table S1). A PHI statistical test was applied to evaluate the significance of recombination evidence between closely and distantly related genomes. Furthermore, the point of recombination along with major and minor parents of the recombinant was accessed through RDP, Bootscan, MaxChi, Chimaera and 3Seq methods [59].

## Figures and Tables

**Table 1:** Wuhan-Hu-1-CoV homology and genetic variations in different genomic regions with respect to SARS-CoV.

Envelop Protein		Membrane Protein		Nucleocapsid Protein		Spike Protein	
Homology	Genetic Variations	Homology	Genetic Variations	Homology	Genetic Variations	Homology	Genetic Variations
93%	07%	92%	08%	93%	07%	81%	19%

[Wuhan-Hu-1-CoV (Wuhan seafood market pneumonia virus) and SARS-CoV (GZ02)].

**Table 2:** Recombination regions identified with position of break and endpoint, and major and minor parents.

Sr.No.	Region	Position of Break and Endpoint		Parents		Methods and *p*-Values				
		Begin	End	Major	Minor	RDP	Bootscan	MaxChi	Chimaera	3Seq
1	RdRp	15504	16692	ZXC21	Rf1	3.1 × 10^−25^	2.9 × 10^−26^	2.8 × 10^−12^	3.2 × 10^−15^	8.7 × 10^−04^
2	Helicase	16693	17932	ZC45	W1V1	4.4 × 10^−13^	1.6 × 10^−12^	2.3 × 10^−02^	7.2 × 10^−11^	-
3	S	22077	22124	ZC45	Rf1	1.8 × 10^−03^	-	-	-	8.5 × 10^−04^
4	S	22299	22435	Rf1	GZ02	1.4 × 10^−02^	-	-	-	3.5 × 10^−30^
5	S	23117	23270	ZXC21	W1V1	1.0 × 10^−05^	3.4 × 10^−05^	-	1.6 × 10^−02^	2.3 × 10^−11^
6	S	23519	23787	ZXC21	Rf1	4.5 × 10^−14^	6.8 × 10^−13^	4.5 × 10^−04^	6.4 × 10^−05^	6.2 × 10^−14^
7	S	23897	24342	ZXC21	Rf1	8.5 × 10^−16^	8.8 × 10^−12^	-	-	-
8	S	24716	24790	ZC45	GZ02	1.8 × 10^−05^	1.4 × 10^−04^	-	-	1.7 × 10^−03^
9	ORF3a	25745	25862	ZC45	GZ02	3.8 × 10^−08^	4.1 × 10^−07^	-	-	1.5 × 10^−04^

[Bat SARS-Like CoVs W1V1, ZXC21, ZC45; Bat SARS-CoVs GZ02, RF1, and MERS-CoV].

## Supplementary Materials

The following are available online at https://www.mdpi.com/2076-0817/9/3/240/s1, Figure S1: Envelop (E) protein multialign sequence comparison of Wuhan-Hu-1-CoV (Wuhan seafood market pneumonia virus), SARS-CoV (GZ02) and MERS CoV, Figure S2: Membrane (M) protein multialign sequence comparison of Wuhan-Hu-1-CoV (Wuhan seafood market pneumonia virus), SARS-CoV (GZ02) and MERS CoV, Figure S3: Nucleocapsid (N) protein multialign sequence comparison of Wuhan-Hu-1-CoV (Wuhan seafood market pneumonia virus), SARS-CoV (GZ02) and MERS CoV, Figure S4: Spike (S) protein multialign sequence comparison of Wuhan-Hu-1-CoV (Wuhan seafood market pneumonia virus), SARS-CoV (GZ02) and MERS Co, Figure S5: Receptor binding domain (306-527) comparison between Wuhan-Hu-1 (Wuhan seafood market pneumonia virus), SARS-CoV (GZ02), Figure S6: Receptor binding motif receptor binding to human ACE2 (424-494); Comparison between Wuhan-Hu-1-CoV (Wuhan seafood market pneumonia virus), SARS-CoV (GZ02),Table S1: Coronavirus and their NCBI accession numbers used for phylogenetic analysis, Table S2: Bayesian information criterion (BIC) values for nucleotides substitution model selection.

## References

[1] Gao G.F. (2018). From “A”IV to “Z”IKV: Attacks from emerging and re-emerging pathogens. Cell.

[2] Zhu N., Zhang D., Wang W., Li X., Yang B., Song J., Niu P. (2020). A novel coronavirus from patients with pneumonia in China, 2019. N. Engl. J. Med..

[3] Gorbalenya E.A., Baker S.C., Baric R.S., Groot R.J., Drosten C., Gulyaeva A.A., Haagmans B.L., Lauber C., Leontovich A.M., Neuman B.W. (2020). Severe acute respiratory syndrome-related coronavirus: The species and its viruses—A statement of the Coronavirus Study Group. bioRxiv.

[4] Masters P.S., Perlman S., Knipe D.M., Howley P.M. (2013). Coronaviridae. Fields Virology.

[5] Weiss S.R., Leibowitz J.L. (2011). Coronavirus pathogenesis. Adv. Virus Res..

[6] Su S., Wong G., Shi W., Liu J., Lai A.C., Zhou J., Gao G.F. (2016). Epidemiology, genetic recombination, and pathogenesis of coronaviruses. Trends Microbiol..

[7] Cui J., Li F., Shi Z.L. (2019). Origin and evolution of pathogenic coronaviruses. Nat. Rev. Microbiol..

[8] Lau S.K., Woo P.C., Li K.S., Huang Y., Tsoi H.W., Wong B.H., Wong S.S., Leung S.Y., Chan K.H., Yuen K.Y. (2005). Severe acute respiratory syndrome coronavirus-like virus in Chinese horseshoe bats. Proc. Natl. Acad. Sci. USA.

[9] WHO (MERS-CoV) (2020). https://www.who.int/emergencies/mers-cov/en/.

[10] WHO (Novel Coronavirus COVID-19 Situation) (2020). https://experience.arcgis.com/experience/685d0ace521648f8a5beeeee1b9125cd.

[11] Lai M.M.C. (1996). Recombination in large RNA viruses: Coronaviruses. Semin. Virol..

[12] Sawicki S.G., Sawicki D.L. (1998). A new model for coronavirus transcription. Adv. Exp. Med. Biol..

[13] Spaan W., Delius H., Skinner M.A., Armstrong J., Rottier P., Smeekens S., Siddell S.G., Zeijst B. (1984). Transcription strategy of coronaviruses: Fusion of non-contiguous sequences during mRNA synthesis. Adv. Exp. Med. Biol..

[14] Marle G., Most R.G., Straaten T., Luytjes W., Spaan W.J. (1995). Regulation of transcription of coronaviruses. Adv. Exp. Med. Biol..

[15] Kottier S.A., Cavanagh D., Britton P. (1995). Experimental evidence of recombination in coronavirus infectious bronchitis virus. Virology.

[16] Cavanagh D., Davis P.J. (1988). Evolution of avian coronavirus IBV: Sequence of the matrix glycoprotein gene and intergenic region of several serotypes. J. Gen. Virol..

[17] Cavanagh D., Davis P.J., Cook J.K.A. (1992). Infectious bronchitis virus: Evidence for recombination within the Massachusetts serotype. Avian Pathol..

[18] Jia W., Karaca K., Parrish C.R., Naqi S.A. (1995). Anovel variant of avian infectious bronchitis virus resulting from recombination among three different strains. Arch. Virol..

[19] Wang L., Junker D., Collisson E.W. (1993). Evidence of natural recombination within the S1 gene of infectious bronchitis virus. Virology.

[20] Wang L., Junker D., Hock L., Ebiary E., Collisson E.W. (1994). Evolutionary implications of genetic variations in the S1 gene of infectious bronchitis virus. Virus Res..

[21] Wang L., Xu Y., Collisson E.W. (1997). Experimental confirmation of recombination upstream of the S1 hypervariable region of infectious bronchitis virus. Virus Res..

[22] Markino S., Keck J.G., Stohlman S.A., Lai M.M.C. (1986). High-frequency RNA recombination of murine coronaviruses. J. Virol..

[23] Wong G., Liu W., Liu Y., Zhou B., Bi Y., Gao G.F. (2015). MERS, SARS, and Ebola: The role of super-spreaders in infectious disease. Cell Host Microbe.

[24] Fan Y., Zhao K., Shi Z.L., Zhou P. (2019). Bat Coronaviruses in China. Viruses.

[25] World Health Organization Novel Coronavirus—Japan (ex-China). https://www.who.int/csr/don/16-january-2020-novel-coronavirus-japan-ex-china/en/.

[26] CDC (2020). 2019 Novel Coronavirus (2019-nCoV), Wuhan, China. https://www.cdc.gov/coronavirus/novel-coronavirus-2019.html.

[27] Hui E.K.W. (2006). Reasons for the increase in emerging and re-emerging viral infectious diseases. Microbes Infect..

[28] Menachery V.D., Yount B.L., Sims A.C., Debbink K., Agnihothram S.S., Gralinski L.E., Swanstrom J. (2016). SARS-like WIV1-CoV poised for human emergence. Proc. Natl. Acad. Sci. USA.

[29] Menachery V.D., Yount B.L., Debbink K., Agnihothram S., Gralinski L.E., Plante J.A., Graham R.L., Scobey T., Ge X.Y., Donaldson E.F. (2015). A SARS-like cluster of circulating bat coronaviruses shows potential for human emergence. Nat. Med..

[30] Rambaut A. (2020). Preliminary Phylogenetic Analysis of 11 nCoV2019 Genomes, 2020-01-19. http://virological.org/t/preliminary-phylogenetic-analysis-of-11-ncov2019-genomes-2020-01-19/329.

[31] Bedford T., Neher R. (2020). Genomic Epidemiology of Novel Coronavirus (nCoV) Using Data Generated by Fudan University, China CDC, Chinese Academy of Medical Sciences, Chinese Academy of Sciences and the Thai National Institute of Health Shared via GISAID. https://nextstrain.org/ncov.

[32] Menachery V.D., Graham R.L., Baric R.S. (2017). Jumping species-a mechanism for coronavirus persistence and survival. Curr. Opin. Virol..

[33] Becker M.M., Graham R.L., Donaldson E.F., Rockx B., Sims A.C., Sheahan T., Pickles R.J., Corti D., Johnston R.E., Baric R.S. (2008). Synthetic recombinant bat SARS-like coronavirus is infectious in cultured cells and in mice. Proc. Natl. Acad. Sci. USA.

[34] Dolan P.T., Whitfield Z.J., Andino R. (2018). Mapping the evolutionary potential of RNA viruses. Cell Host Microbe.

[35] Nao N., Yamagishi J., Miyamoto H., Igarashi M., Manzoor R., Ohnuma A., Kishida N. (2017). Genetic predisposition to acquire a polybasic cleavage site for highly pathogenic avian influenza virus hemagglutinin. MBio.

[36] Chan C.M., Woo P.C., Lau S.K., Tse H., Chen H.L., Li F., Yuen K.Y. (2008). Spike protein, S, of human coronavirus HKU1: Role in viral life cycle and application in antibody detection. Exp. Biol. Med..

[37] Follis K.E., York J., Nunberg J.H. (2008). Furin cleavage of the SARS coronavirus spike glycoprotein enhances cell–cell fusion but does not affect virion entry. Virology.

[38] Menachery V.D., Dinnon K.H., Yount B.L., McAnarney E.T., Gralinski L.E., Hale A., Graham B. (2020). Trypsin Treatment Unlocks Barrier for Zoonotic Bat Coronavirus Infection. J. Virol..

[39] Alexander D.J., Brown I.H. (2009). History of highly pathogenic avian influenza. Rev. Sci. Tech..

[40] Peng Z., Xing-Lou Y., Xian-Guang W. (2020). A pneumonia outbreak associated with a new coronavirus of probable bat origin. Nature.

[41] Wu F., Zhao S., Yu B. (2020). A new coronavirus associated with human respiratory disease in China. Nature.

[42] Domingo E., Biebrichen C.K., Eigen M., Holland J.J. (2001). Quasispecies and RNA Virus Evolution: Principles and Consequences.

[43] Consortium T.C.S. (2004). Molecular evolution of the SARS coronavirus during the course of the SARS epidemic in China. Science.

[44] Xu X., Chen P., Wang J., Feng J., Zhou H., Li X., Zhong W., Hao P. (2020). Evolution of the novel coronavirus from the ongoing Wuhan outbreak and modeling of its spike protein for risk of human transmission. Sci. China Life Sci..

[45] Pradhan P., Pandey A.K., Mishra A., Gupta P., Tripathi P.K., Menon M.B., Kundu B. (2020). Uncanny similarity of unique inserts in the 2019-nCoV spike protein to HIV-1 gp120 and Gag. bioRxiv.

[46] Worobey M., Holmes E.C. (1999). Evolutionary aspects of recombination in RNA viruses. J. Gen. Virol..

[47] Lai M.M.C. (1992). RNA recombination in animal and plant viruses. Microbiol. Rev..

[48] Lai M.M.C., Cavanagh D. (1997). The molecular biology of coronaviruses. Adv. Virus Res..

[49] Jackwood M.W., Boynton T.O., Hilt D.A., McKinley E.T., Kissinger J.C., Paterson A.H., Robertson J., Lemke C., McCall A.W., Williams S.M. (2010). Emergence of a group 3 coronavirus through recombination. Virology.

[50] Posada D. (2002). Evaluation of methods for detecting recombination from DNA sequences: Empirical data. Mol. Biol. Evol..

[51] Stavrinides J., Guttman D.S. (2004). Mosaic evolution of the severe acute respiratory syndrome coronavirus. J. Virol..

[52] Zhang X.W., Yap Y.L., Danchin A. (2004). Testing the hypothesis of a recombinant origin of the SARS-associated coronavirus. Arch. Virol..

[53] Kazutaka K., John R., Kazunori D.Y. (2019). MAFFT online service: Multiple sequence alignment, interactive sequence choice and visualization. Brief. Bioinform..

[54] Darriba D., Taboada G.L., Doallo R., Posada D. (2012). jModelTest 2: More models, new heuristics and parallel computing. Nat. Methods.

[55] Rambaut A. (2010). FigTree v1.4.4. Institute of Evolutionary Biology.

[56] Corpet F. (1988). Multiple sequence alignment with hierarchical clustering. Nucleic Acids Res..

[57] Kelley L.A., Mezulis S., Yates C.M., Wass M.N., Sternberg M.J. (2015). The Phyre2 web portal for protein modeling, prediction and analysis. Nat. Protoc..

[58] DeLano W.L. (2002). The PyMOL Molecular Graphics System, Version 1.1.

[59] Martin D.P., Williamson C., Posada D. (2005). RDP2: Recombination detection and analysis from sequence alignments. Bioinformatics.

[60] Lole K.S., Bollinger R.C., Paranjape R.S., Gadkari D., Kulkarni S.S., Novak N.G., Ingersoll R., Sheppard H.W., Ray S.C. (1999). Full-length human immunodeficiency virus type 1 genomes from subtype C-infected seroconverters in India, with evidence of intersubtype recombination. J. Virol..

