# Supplementary material for: Evolutionary Trajectory for the Emergence of Novel Coronavirus SARS-CoV-2
Source: Pathogens. 2020 Mar 23;9(3):240. doi: 10.3390/pathogens9030240 (PMC7157669; doi:10.3390/pathogens9030240)
Supplement: Supplementary file 1 [file pathogens-09-00240-s001.pdf]

pathogens

MDPI

Article

Evolutionary Trajectory for the Emergence of Novel Coronavirus SARS-CoV-2

Saif ur Rehman [1], Laiba Shafique [1], Awais Ihsan [2,3,*] and Qingyou Liu [1,*]

[1] State Key Laboratory for Conservation and Utilization of Subtropical Agro-bioresources of Guangxi University, Nanning 530005, China; Saifurrehman4337904@gmail.com (S.u.R.), laibazoologist@gmail.com (L.A.)
[2] Department of Biosciences, COMSATS University Islamabad, Sahiwal Campus 57000, Pakistan
[3] College of Pharmacy, South Central University for Nationalities, Wuhan 430074, China
* Correspondence: awais.dr@gmail.com (A.I.) and qyliu-gene@gxu.edu.cn (Q.L.); Tel.: +8613878805296

Received: 3 March 2020; Accepted: 21 March 2020; Published: 23 March 2020

Table S1. Coronavirus and their NCBI accession numbers used for phylogenetic analysis.

NCBI accession number	Strain	Full Name
NC_038861.1	TGEV Purdue	Transmissible gastroenteritis virus
DQ811787.1	PRCV ISU-1	porcine respiratory coronavirus
DQ848678.1	FCoV C1Je	Feline coronavirus
NC_005831.2	HCoV-NL63	Human Coronavirus NL63
NC_002645.1	HCoV-229E	Human coronavirus 229E
EU420139.1	Mi-BatCoV AFCD77	Miniopterus bat coronavirus
EU420137.1	Mi-BatCoV 1B AFCD307	Bat coronavirus 1B strain AFCD307
NC_010437.1	Mi-BatCoV 1A AFCD62	Bat coronavirus 1A
NC_018871.1	Ro-BatCoV HKU10	Rousettus bat coronavirus HKU10
NC_022103.1	BatCoV CDPHE15/USA/2006	BatCoV CDPHE15/USA/2006
NC_009657.1	Sc BatCoV 512/2005	Scotophilus bat coronavirus
AF353511.1	PEDV CV777	Porcine epidemic diarrhea virus strain CV777
KU131570.1	HCoV-OC43	Human coronavirus OC43 strain
DQ011855.1	PHEV VW572	Porcine hemagglutinating encephalomyelitis virus
KT444582.1	SARS-like_coronavirus_WIV16	
MH940245.1	HCoV-HKU1	Human coronavirus HKU1
NC_001846.1	MHV-A59	Mouse hepatitis virus strain MHV-A59 C12
KF367457.1	Bat SARSr-CoV WIV1 Rhinolophus sinicus	Bat SARS-like coronavirus WIV1
DQ071615.1	Bat SARSr-CoV Rp3	Bat SARS coronavirus Rp3
DQ022305.2	Bat SARSr-CoV HKU3-1	Bat SARS coronavirus HKU3
DQ412043.1	Bat SARSr-CoV Rm1 Rhinolophus macrotis	Bat SARS coronavirus Rm1
DQ412042.1	Bat SARSr-CoV Rf1	Bat SARS coronavirus Rf1
AY508724.1	SARS coronavirus NS-1	SARS coronavirus NS-1
AY485277	SARS_coronavirus_Sino1-11	SARS_coronavirus_Sino1-11
AY390556	SARS_coronavirus_GZ02	SARS_coronavirus_GZ02
AY278489	SARS_coronavirus_GD01	SARS_coronavirus_GD01
DQ648794.1	BtCoV/133/2005	Bat coronavirus V/133/2005
MH734115.1	MERS-CoV	Middle East respiratory syndrome-related coronavirus

EF065513.1	BtCoV HKU9-1	Bat coronavirus HKU9-1
EF065516.1	BatCoV HKU9-4	Bat coronavirus HKU9-4
EF065515.1	BatCoV HKU9-3	Bat coronavirus HKU9-3
EF065514.1	BatCoV HKU9-2	Bat coronavirus HKU9-2
MG772934.1	Bat-SL-CoVZXC21	Bat SARS-like coronavirus
MG772933.1	Bat-SL-CoVZC45	Bat SARS-like coronavirus
NC_016994.1	Night-heron CoV HKU19	Night-heron coronavirus HKU19
NC_016995.1	Wigeon CoV HKU20	Wigeon coronavirus HKU20
NC_016996.1	Common-moorhen CoV HKU21	Common-moorhen coronavirus HKU21
NC_011550.1	Munia CoV HKU13-3514	Munia coronavirus HKU13-3514
NC_016991.1	White-eye CoV HKU16	White-eye coronavirus HKU16
NC_011549.1	Thrush CoV HKU12-600	Thrush coronavirus HKU12-600
NC_011547.1	Bulbul CoV HKU11-934	Bulbul coronavirus HKU11-934
NC_010646.1	BWCoV SW1	Beluga Whale coronavirus SW1
AJ311317.1_	IBV_Beaudette_CK	Avian infectious bronchitis virus Beaudette
NC_045512.2	Wuhan-Hu-1	Wuhan seafood market pneumonia virus isolate Wuhan-Hu-1
MN988668.1	2019-nCoV WHU01	Wuhan seafood market pneumonia virus isolate 2019-nCoV WHU01
MN988669	2019-nCoV WHU02	Wuhan seafood market pneumonia virus isolate 2019-nCoV WHU02
MN988713	2019-nCoV/USA-IL1/2020	Wuhan seafood market pneumonia virus isolate
MN997409	2019-nCoV/USA-AZ1/2020	Wuhan seafood market pneumonia virus isolate 2019-nCoV/USA-AZ1/2020
MN985325.1	2019-nCoV/USA-WA1/2020	Wuhan seafood market pneumonia virus isolate 2019-nCoV/USA-WA1/2020
MN975262	2019-nCoV_HKU-SZ-005b_2020	Wuhan seafood market pneumonia virus isolate 2019-nCoV_HKU-SZ-005b_2020
MN938384	2019-nCoV_HKU-SZ-002a_2020	Wuhan seafood market pneumonia virus isolate 2019-nCoV_HKU-SZ-002a_2020
NMDC60013002-05	BetaCoVWuhanWH190022019	Wuhan seafood market pneumonia virus BetaCoV / Wuhan / WH19002 / 2019
NMDC60013002-07	BetaCoV / Wuhan / YS8011 / 2020	Wuhan seafood market pneumonia virus

Table S2. Bayesian information criterion (BIC) values for nucleotides substitution model selection.

ID	NAME	PARTITION	-1IL	P	BIC	DELTA BIC	WEIGHT	CUM WEIGHT	uDELTA
1	JC	000000	914100.5795	104	1829325.4632	99383.3515	0.0	1.0	-
2	JC+I	000000	899077.0642	105	1799289.2432	69347.1316	0.0	1.0	-
3	JC+G	000000	888595.5907	105	1778326.2962	48384.1846	0.0	1.0	-
4	JC+I+G	000000	886292.7187	106	1773731.3627	43789.251	0.0	1.0	-
5	F81	000000	911097.9293	107	1823352.5945	93410.4828	0.0	1.0	-
6	F81+I	000000	895781.524	108	1792730.5947	62788.483	0.0	1.0	-
7	F81+G	000000	883022.6587	108	1767212.864	37270.7523	0.0	1.0	-
8	F81+I+G	000000	881099.889	109	1763378.1353	33436.0236	0.0	1.0	-
9	K80	010010	904187.4933	105	1809510.1014	79567.9897	0.0	1.0	-
10	K80+I	010010	888910.6315	106	1778967.1883	49025.0766	0.0	1.0	-
11	K80+G	010010	876796.3837	106	1754738.6927	24796.5811	0.0	1.0	-
12	K80+I+G	010010	874722.137	107	1750601.01	20658.8983	0.0	1.0	-
13	HKY	010010	898982.1874	108	1799131.9215	69189.8098	0.0	1.0	-
14	HKY+I	010010	883187.0687	109	1767552.4946	37610.3829	0.0	1.0	-
15	HKY+G	010010	867361.7698	109	1735901.8967	5959.7851	0.0	1.0	-
16	HKY+I+G	010010	865687.0489	110	1732563.2655	2621.1539	0.0	1.0	-
17	TrNef	010020	904164.502	106	1809474.9293	79532.8177	0.0	1.0	-
18	TrNef+I	010020	888890.5198	107	1778937.7755	48995.6638	0.0	1.0	-
19	TrNef+G	010020	876792.3255	107	1754741.387	24799.2754	0.0	1.0	-
20	TrNef+I+G	010020	874720.6069	108	1750608.7603	20666.6487	0.0	1.0	-

21	TrN	010020	898753.7097	109	1798685.7766	68743.665	0.0	1.0	-
22	TrN+I	010020	882984.8821	110	1767158.932	37216.8204	0.0	1.0	-
23	TrN+G	010020	867342.3046	110	1735873.7771	5931.6655	0.0	1.0	-
24	TrN+I+G	010020	865662.3268	111	1732524.632	2582.5204	0.0	1.0	-
33	TPM2	010212	901552.7593	106	1804251.4439	74309.3323	0.0	1.0	-
34	TPM2+I	010212	886422.3629	107	1774001.4618	44059.3501	0.0	1.0	-
35	TPM2+G	010212	874274.2676	107	1749705.2712	19763.1596	0.0	1.0	-
36	TPM2+I+G	010212	872235.5535	108	1745638.6537	15696.542	0.0	1.0	-
37	TPM2uf	010212	897935.8956	109	1797050.1483	67108.0367	0.0	1.0	-
38	TPM2uf+I	010212	882320.5431	110	1765830.254	35888.1424	0.0	1.0	-
39	TPM2uf+G	010212	866771.3515	110	1734731.8708	4789.7592	0.0	1.0	-
40	TPM2uf+I+G	010212	865089.2863	111	1731378.5509	1436.4393	0.0	1.0	-
57	TIM2ef	010232	901535.9953	107	1804228.7267	74286.615	0.0	1.0	-
58	TIM2ef+I	010232	886407.6797	108	1773982.906	44040.7943	0.0	1.0	-
59	TIM2ef+G	010232	874277.0652	108	1749721.677	19779.5653	0.0	1.0	-
60	TIM2ef+I+G	010232	872235.2887	109	1745648.9347	15706.8231	0.0	1.0	-
61	TIM2	010232	897707.2895	110	1796603.7469	66661.6352	0.0	1.0	-
62	TIM2+I	010232	882119.009	111	1765437.9965	35495.8849	0.0	1.0	-
63	TIM2+G	010232	866738.831	111	1734677.6405	4735.5289	0.0	1.0	-
64	TIM2+I+G	010232	865052.3642	112	1731315.5175	1373.4059	0.0	1.0	-
41	TPM3	012012	903440.6626	106	1808027.2505	78085.1388	0.0	1.0	-
42	TPM3+I	012012	888161.5517	107	1777479.8395	47537.7278	0.0	1.0	-
43	TPM3+G	012012	876546.5123	107	1754249.7605	24307.6489	0.0	1.0	-
44	TPM3+I+G	012012	874426.1422	108	1750019.831	20077.7193	0.0	1.0	-
45	TPM3uf	012012	899047.6978	109	1799273.7528	69331.6412	0.0	1.0	-
46	TPM3uf+I	012012	883246.9078	110	1767682.9834	37740.8718	0.0	1.0	-
47	TPM3uf+G	012012	866551.9145	110	1734292.9968	4350.8851	0.0	1.0	-
48	TPM3uf+I+G	012012	865016.611	111	1731233.2003	1291.0887	0.0	1.0	-
65	TIM3ef	012032	903472.0542	107	1808100.8443	78158.7327	0.0	1.0	-
66	TIM3ef+I	012032	888147.807	108	1777463.1605	47521.0489	0.0	1.0	-
67	TIM3ef+G	012032	876546.7924	108	1754261.1313	24319.0197	0.0	1.0	-
68	TIM3ef+I+G	012032	874423.1419	109	1750024.6411	20082.5294	0.0	1.0	-
69	TIM3	012032	898812.7423	110	1798814.6524	68872.5408	0.0	1.0	-
70	TIM3+I	012032	883045.0929	111	1767290.1643	37348.0527	0.0	1.0	-
71	TIM3+G	012032	866501.6048	111	1734203.1881	4261.0765	0.0	1.0	-
72	TIM3+I+G	012032	864966.0137	112	1731142.8165	1200.7049	0.0	1.0	-
25	TPM1	012210	903618.7681	106	1808383.4615	78441.3498	0.0	1.0	-
26	TPM1+I	012210	888253.1842	107	1777663.1044	47720.9927	0.0	1.0	-
27	TPM1+G	012210	876361.5225	107	1753879.781	23937.6694	0.0	1.0	-
28	TPM1+I+G	012210	874243.2697	108	1749654.0859	19711.9743	0.0	1.0	-
29	TPM1uf	012210	898952.3011	109	1799082.9595	69140.8478	0.0	1.0	-
30	TPM1uf+I	012210	883158.0742	110	1767505.3162	37563.2046	0.0	1.0	-
31	TPM1uf+G	012210	867361.3015	110	1735911.7708	5969.6592	0.0	1.0	-
32	TPM1uf+I+G	012210	865683.9287	111	1732567.8359	2625.7243	0.0	1.0	-
49	TIM1ef	012230	903595.3084	107	1808347.3528	78405.2411	0.0	1.0	-
50	TIM1ef+I	012230	888232.8571	108	1777633.2608	47691.1491	0.0	1.0	-
51	TIM1ef+G	012230	876356.3963	108	1753880.3391	23938.2275	0.0	1.0	-
52	TIM1ef+I+G	012230	874243.8793	109	1749666.1159	19724.0043	0.0	1.0	-
53	TIM1	012230	898723.3885	110	1798635.9448	68693.8332	0.0	1.0	-
54	TIM1+I	012230	882957.176	111	1767114.3303	37172.2187	0.0	1.0	-
55	TIM1+G	012230	867341.2268	111	1735882.4321	5940.3205	0.0	1.0	-
56	TIM1+I+G	012230	865658.9064	112	1732528.6019	2586.4903	0.0	1.0	-
73	TVMef	012314	900683.8853	108	1802535.3173	72593.2056	0.0	1.0	-
74	TVMef+I	012314	885552.4015	109	1772283.1602	42341.0485	0.0	1.0	-
75	TVMef+G	012314	873957.4473	109	1749093.2518	19151.1402	0.0	1.0	-
76	TVMef+I+G	012314	871860.5101	110	1744910.188	14968.0764	0.0	1.0	-
77	TVM	012314	897934.6949	111	1797069.3683	67127.2567	0.0	1.0	-
78	TVM+I	012314	882318.388	112	1765847.5649	35905.4533	0.0	1.0	-
79	TVM+G	012314	865961.8492	112	1733134.4873	3192.3757	0.0	1.0	-
80	TVM+I+G	012314	864420.9661	113	1730063.5319	121.4203	0.0	1.0	-
81	SYM	012345	900706.0847	109	1802590.5266	72648.415	0.0	1.0	-
82	SYM+I	012345	885546.4423	110	1772282.0524	42339.9408	0.0	1.0	-
83	SYM+G	012345	873956.7992	110	1749102.7662	19160.6546	0.0	1.0	-
84	SYM+I+G	012345	871860.2261	111	1744920.4307	14978.3191	0.0	1.0	-
85	GTR	012345	897658.6512	112	1796528.0914	66585.9798	0.0	1.0	-
86	GTR+I	012345	882074.5856	113	1765370.7709	35428.6593	0.0	1.0	-
87	GTR+G	012345	865904.1275	113	1733029.8546	3087.7429	0.0	1.0	-
88	GTR+I+G	012345	864354.8507	114	1729942.1116	0.0	1.0	1.0	-

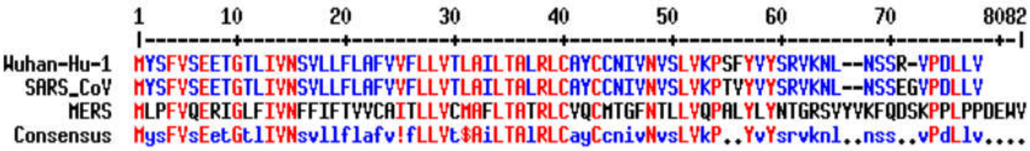

Figure S1. Envelop (E) protein multialign sequence comparison of Wuhan-Hu-1-CoV (Wuhan seafood market pneumonia virus), SARS-CoV (GZ02) and MERS CoV.

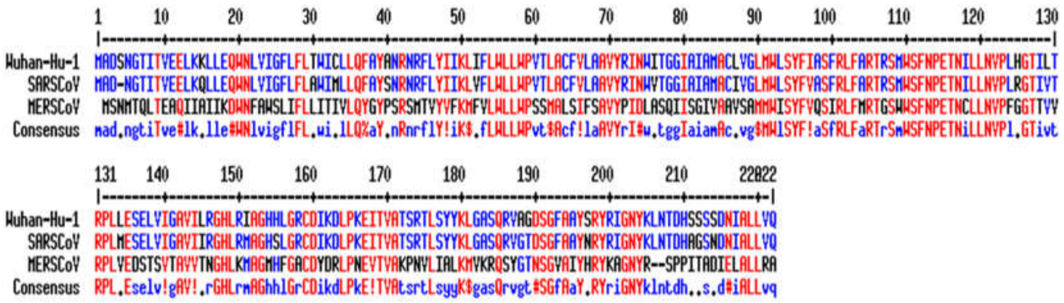

Figure S2. Membrane (M) protein multialign sequence comparison of Wuhan-Hu-1-CoV (Wuhan seafood market pneumonia virus), SARS-CoV (GZ02) and MERS CoV.

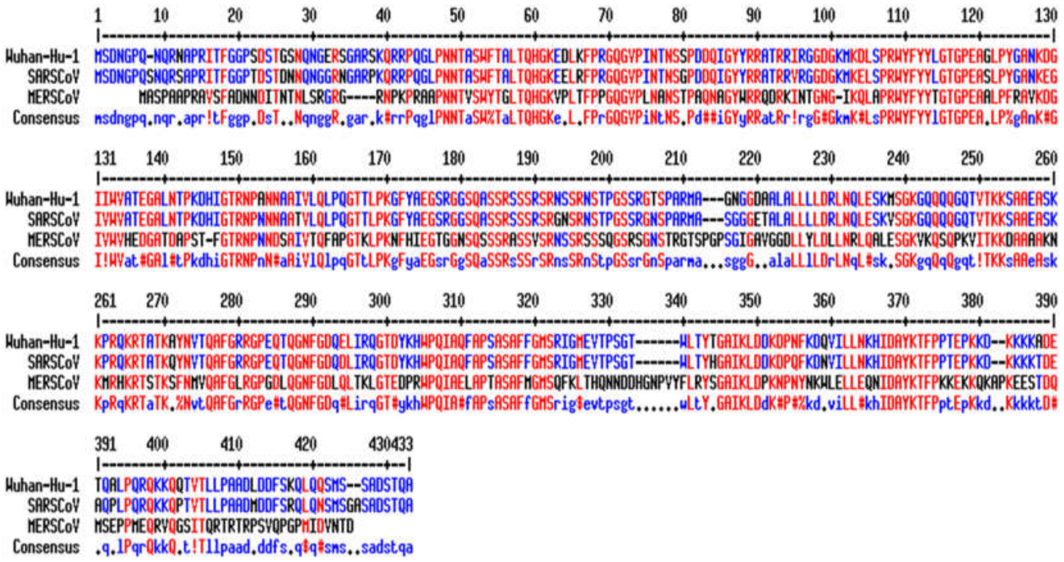

Figure S3. Nucleocapsid (N) protein multialign sequence comparison of Wuhan-Hu-1-CoV (Wuhan seafood market pneumonia virus), SARS-CoV (GZ02) and MERS CoV.

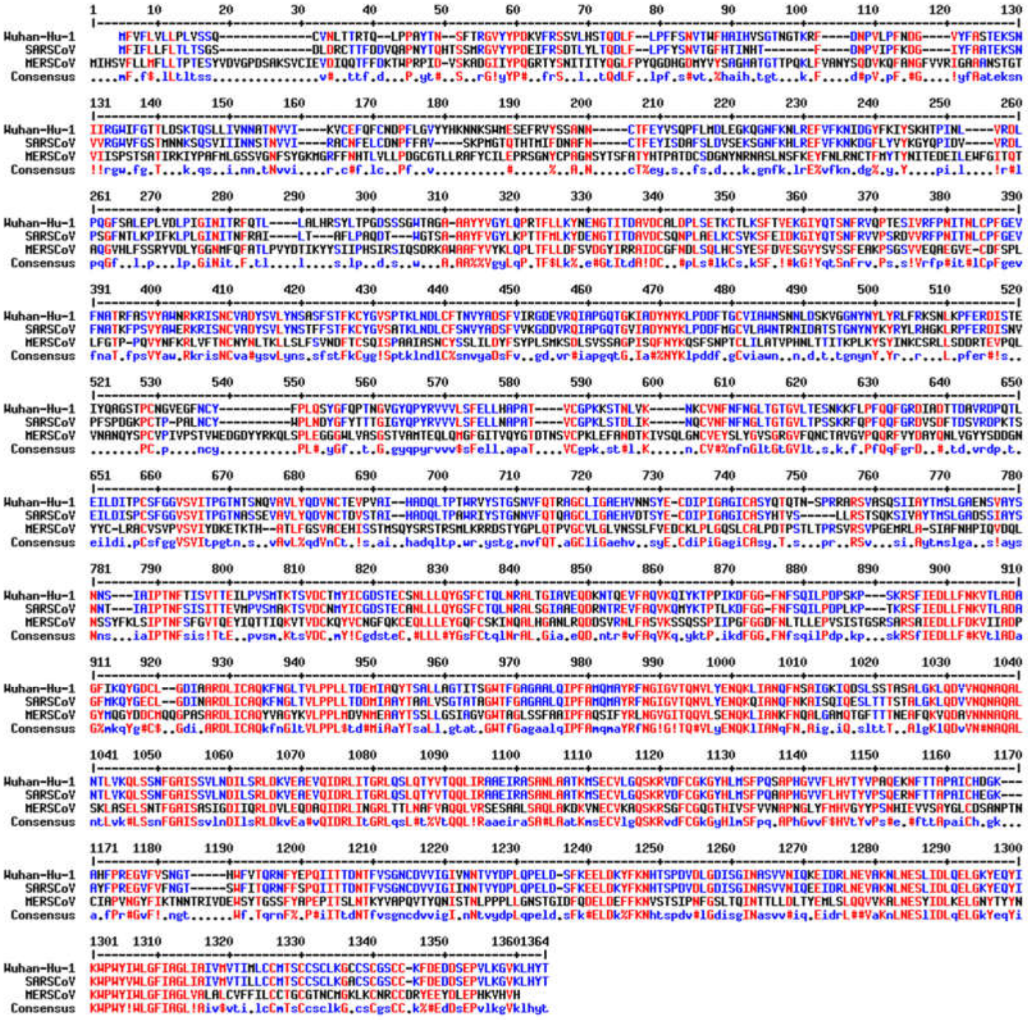

Figure S4. Spike (S) protein multialign sequence comparison of Wuhan-Hu-1-CoV (Wuhan seafood market pneumonia virus), SARS-CoV (GZ02) and MERS CoV.

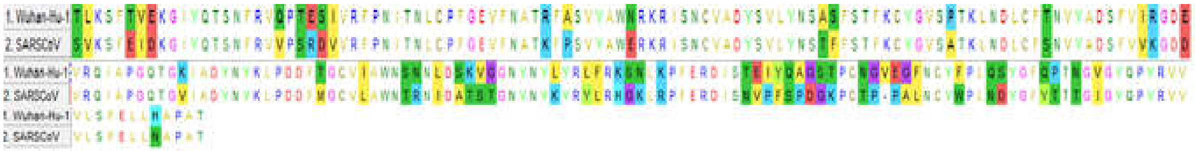

Figure S5. Receptor binding domain (306-527) comparison between Wuhan-Hu-1 (Wuhan seafood market pneumonia virus), SARS-CoV (GZ02).

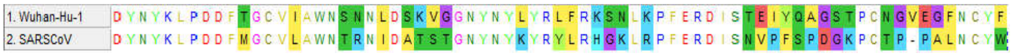

Figure S6. Receptor binding motif receptor binding to human ACE2 (424-494); Comparison between Wuhan-Hu-1-CoV (Wuhan seafood market pneumonia virus), SARS-CoV (GZ02).

© 2020 by the authors. Submitted for possible open access publication under the terms and conditions of the Creative Commons Attribution (CC BY) license (http://creativecommons.org/licenses/by/4.0/).
